# The Effect of Body Mass on the Shoe-Athlete Interaction

**DOI:** 10.1155/2017/7136238

**Published:** 2017-03-29

**Authors:** A. Tsouknidas, M. Pantazopoulos, D. Sagris, D. Fasnakis, S. Maropoulos, F. Arabatzi, N. Michailidis

**Affiliations:** ^1^Department of Mechanical Engineering, Aristotle University of Thessaloniki, 54 124 Thessaloniki, Greece; ^2^Department of Mechanical Engineering, Technical University of Central Macedonia, Terma Magnisias, 62 124 Serres, Greece; ^3^Department of Mechanical Engineering, Technical University of Western Macedonia, Kila, 50 100 Kozani, Greece; ^4^School of Physical Education & Sport Science, Aristotle University of Thessaloniki, Ippokratous 22 Ag. Ioannis, 62 122 Serres, Greece

## Abstract

Long-distance running is known to induce joint overloading and elevate cytokine levels, which are the hallmarks for a variety of running-related injuries. To address this, footwear systems incorporate cushioning midsoles to mitigate injurious mechanical loading. The aim of this study was to evaluate the effect of athlete body mass on the cushioning capacity of technical footwear. An artificial heel was prototyped to fit the impact pattern of a heel-strike runner and used to measure shock attenuation by an automated drop test. Impact mass and velocity were modulated to simulate runners of various body mass and speeds. The investigation provided refined insight on running-induced impact transmission to the human body. The examined midsole system was optimized around anthropometric data corresponding to an average (normal) body mass. The results suggest that although modern footwear is capable of attenuating the shock waves occurring during foot strike, improper shoe selection could expose an athlete to high levels of peak stress that could provoke an abnormal cartilage response. The selection of a weight-specific cushioning system could provide optimum protection and could thus prolong the duration of physical exercise beneficial to maintaining a simulated immune system.

## 1. Introduction

Human locomotion is a self-optimizing activity [1] as our neuromuscular system constantly adapts to environmental stimuli, with individual gait patterns strongly depending on performer- and environment-specific characteristics (e.g., anatomy, body mass, physical condition, and terrain quality). Lieberman et al. [2] showed that the material characteristics of shoe soles alter the gait of individuals when using minimalist versus cushioned footwear. These changes are based predominantly on the perception of transient shock waves experienced during foot strike, which our body identifies as a source of potential injuries. Based on our experience and perceptual abilities, we thus rapidly adjust our running style to minimize impact during each stride when using different athletic footwear [3]. This subconscious response is based both on how we perceive impact attenuation of a midsole system and on the energy-dissipating properties of the ground [4].

Recent literature [5] emphasizes the importance of environmental invariants which are extracted by the nervous system over time, suggesting that locomotion is a controlled response to environmental cues, and thus, the performer and environment are coparticipants in any resulting action. The very purpose of athletic footwear is to alter the perception of the environment [6] in favour of the performer.

The optimal footwear choice however is subjective, as the way we identify comfort varies significantly among individuals [7]. This choice is generally influenced by preferences developed over time or by the morphological characteristics of an individual's foot [8]—criteria which are however not necessarily bound to shoe quality and performance. Kim et al. [9] argue that leg stiffness strongly depends on the running environment and that footwear systems cannot but be considered an integral part of this performer-environment system.

As a periodic motion, running generates transient forces that measure up to 2.5 times the athlete's body mass. The repetitive nature of these impulses, transmitted through our musculoskeletal system, if within a physiological range, maintains joint homeostasis. Overloading however induces biomechanical and compositional changes in joint tissue [10]. Changes that can cause cell apoptosis followed by collagen degeneration [11]. These stressors, common to long-distance running, have also been linked to increased proinflammatory cytokine levels, for example, TNF-a, which induce a pattern of immunological responses similar to injurious trauma and/or sepsis [12]. With a prevalence of 1 injury for every 1000 hours of training [13], the fine line between physiological and strenuous running could well depend on how athletic footwear cushions generated impulsive forces.

Over the past decade, several studies sought to determine impact attenuation of shoes through the evaluation of force platform measurements [14] and accelerometers mounted on individual test participants [15]. Literature however points out methodological flaws of “runner-inclusive” experimentation, mainly associated with the absorbed energy allocation [16].

ASTM standards suggest the evaluation of running shoes through guided impacts ranging from 5 to 7 joules (ASTM F1614–99, [17]; ASTM F1976–06, [18]). Even though it is adequate to evaluate the shock absorption capacity of athletic footwear destined for a 50-percentile male (175 cm height and 78 kg body mass) running at a high pace [19], it is not sufficient to cover a wide spectrum of subelite runners. According to literature, a 50-percentile male should have a shoe size of 42 [20], but anthropometric data suggest that this shoe size would also cover individuals varying by more than ±30 kg in body mass. This indicates that the impact energy would exceed or fall short of the test limits by more than 40%, an error that might increase even further if gender-specific criteria are considered.

The primary hypothesis of this investigation is that the capacity of a shoe, to attenuate the shock waves developing during running, is strongly related to the impact energy, and thus, each shoe type can only be optimized for a specific body mass and/or impact velocity range. Should this hold true, then shoe selection could become an incremental criterion in predicting joint overloading during running. This would be of high interest to millions of recreational runners, as strenuous training is known to have catabolic effects for cartilaginous components and joint loads severely depend on the impact transferred to the runner during the stance phase [21].

Several studies have sought to address the importance of shoe selection for specific athlete [22] or patient groups [23]. To the best of the authors' knowledge, however, the effect of body mass on the impact attenuating properties of technical footwear has not been documented yet. This study provides a methodology for the systematic evaluation of the mechanical response of athletic footwear to individual runner characteristics (body mass and impact speed), thus granting refined insight into the shock attenuation properties and stability provided by athletic footwear.

## 2. Experimental Methods

As the aim of this investigation was to determine the body mass-dependent impact attenuation properties of athletic footwear, only the conditions occurring at the end of the swing phase (foot strike) were simulated. The experimental procedure considered boundary conditions imitating a heel-strike and normal pronation, as 88.9% of long-distance runners are inclined towards heel-strike patterns irrespective to what their foot strike would be over shorter distances [24].

**Figure 1:**
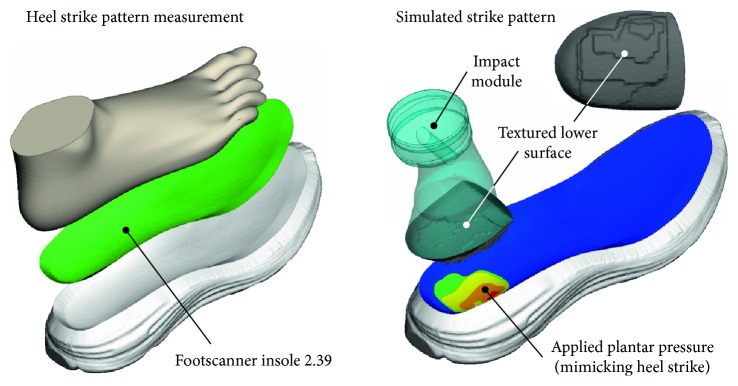
Prototyped impact module recreating the strike pattern of a typical long-distance runner.

To accommodate this consideration during the experiments, the plantar pressure distribution of a heel-strike athlete during the support phase was determined by a Footscanner insole 2.39 system (Niceville, FL 32578, USA) with a 500 Hz sampling rate. A polymer (ABS) heel was prototyped on an open source 3D printer (MendelMax 2) to match both the plantar pressure and the impact angle measured during foot strike [25]. This was achieved by texturing the impact module's lower surface (see the upper-right part of Figure 1).

The protrusion height of the layered texture on the bottom of the artificial heel was evaluated with the Footscanner insole 2.39 system under impact. These protrusions were altered continuously until reproducing a pressure distribution equivalent to the measured one, thus avoiding excess pressures [26], ensuring an optimum simulation of the impact conditions. The edges of the impact module were rounded by a radius of at least 1.2 mm to prevent adverse specimen tearing, with a 4265 mm^2^ surface area delivering the impact.

**Figure 2:**
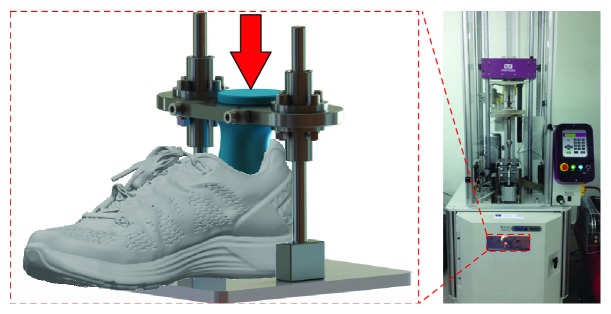
Determination of body mass and strike velocity dependent shock absorption.

The shoe-heel assembly was mounted on a guidance unit, providing rigid support to the shoe while ensuring the application of the impact load in the desired direction. The assembly consisted of two linear rails, guiding the axial ball bearings on which the heel was mounted. This driving structure was supported by an inclined base, resulting in the assembly shown in Figure 2.

The experimental device, along with one shoe specimen each time, was inserted into a modulated INSTRON CEAST 9350, facilitating the measurement of the shock attenuation provided by the shoe. The load, corresponding to a runner body mass range of 45–70 kg, was propagated on the midsole through the artificial heel. The procedure used is in line with a standardized drop weight impact test (Procedure A of ASTM F1614). The mass of the heel-guidance assembly, placed between the force transducer and the shoe, was considered during the drop test calibration to avoid interference with the force and acceleration data.

Three tests were conducted, for statistical purposes, on each of the 3 identical footwear systems (EUR 38 in size) with a gel-based midsole cushioning. All specimens were new, as prior impact conditioning would significantly alter the shock attenuation capacity of footwear. Prior to evaluating their resilience characteristics, by a fully controlled gravity-driven impact of 3.6 to 8.75 J, all shoes were preconditioned by cyclic impact loading. The force application rate and peak displacement were recorded during both loading and unloading cycles. The tests were designed to simulate running conditions corresponding to a female runner of average body mass, while 5 kg impact load variations (ranging from 45 to 70 kg) were also considered to cover under- and overweight runners.

Finally, as the average vertical heel-strike speed [19] varies by ±20%, two reference impulse values were considered to generate compressive forces comparable to various running conditions and athlete heights. The impact velocities during testing corresponded therefore to 0.4 and 0.5 m/s, respectively.

Several parameters were registered during each test. The onshoe (cushioned) impact force was registered along with the maximum vertical displacement and the % force attenuation. The peak displacement values recorded were then used to calculate the average/peak strain and to determine the stability of the shoe in a secondary in situ measurement.

**Figure 3:**
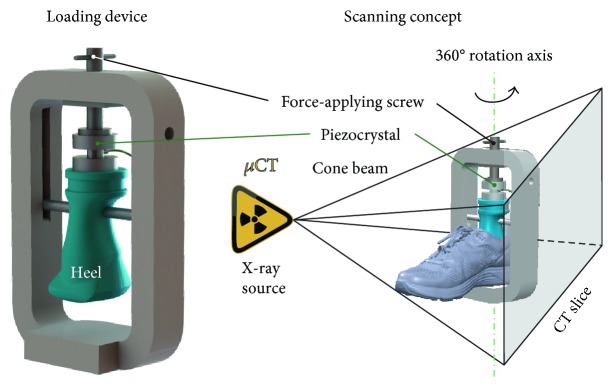
Experimental setup for the evaluation of structural support provided by the shoe.

The strain values (average/peak) were calculated considering both the morphological characteristics of the artificial heel and midsole thickness (varying over the impact surface). To determine the stability, the heel was mounted on a polymer construction (as demonstrated in Figure 3) facilitating the application of loads imitating the displacement values recorded during the impact test, while using the same artificial heel. The loaded construction was scanned by *μ*CT to evaluate the three-dimensional weight-induced insole deformation, thus providing valuable insight on the stability provided by the examined footwear system.

The X-ray apparatus (Werth TomoScope^®^ HV Compact-225 3D CNC) had a 5 *μ*m focal spot, reflection target X-ray tube source, and a digital sensor with an analysis of 1024 × 1024 pixels, operating in the absorption mode to acquire the 2D images of the test device and specimen. Segmentation of the 2D scans resulted in the outline of the shoe's main components (midsole, insole, etc.) in a given cross section, and the 3D data set was generated by overlaying consecutive measurements.

In order to obtain the maximum accuracy during the measurement, the specimens' region of interest was placed exactly on the rotation axis of the table. To obtain comparable results, all the measurement parameters were kept unaltered while changing the applied load up to the predefined vertical displacement values. The distance between the object and the X-ray source, determining the magnification of the measured specimen, was tuned for maximum analysis. The magnification (200 L) resulted in a voxel of a 200 *μ*m side length. The magnitudes of intensity (current) and frequency (voltage) of the X-ray source were selected after several tests and set to 500 *μ*A and 120 kV, respectively, leading to an X-ray power of 60 W. This X-ray power was considered to fit the material density of both the midsole and the loading device.

The reconstruction of the 3D object by means of three processing steps (data preparation, filtering, and back projection) requires several 2D radiographic images (recorded over a 100 ms exposure time) of different orientations over a 360° rotation of the object. A total of 1600 rotational steps ensured a high-quality 3D reconstruction of the shoe, while 4 radiographic images were taken for each orientation.

The measurements were converted to 3D volumetric data by means of WinWerth software and further processed in VG Studio Max. To determine the stability, displacements corresponding to the transverse and longitudinal axis of the shoe were catalogued.

## 3. Results

**Figure 4:**
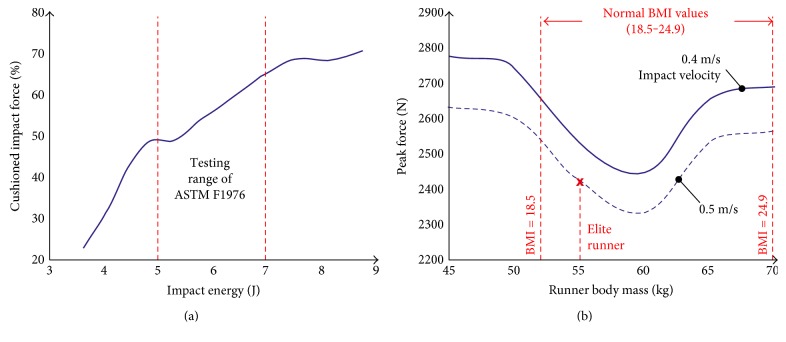
(a) % cushioned force as a function of impact energy. (b) Body mass and strike velocity dependent impulse.

The percentage of the cushioned force versus the impact energy, applied on the tested midsole system under realistic striking conditions (similar to that for heel strike during running), is shown in Figure 4(a). The test range defined by ASTM F1976–06 is outlined by the dashed vertical lines at 5 and 7 J. The information provided by this diagram is difficult to evaluate as to the efficiency of the shoe to mediate the transient shock wave during foot strike. It is however noteworthy that the shock attenuation properties of the midsole material do not correlate linearly to the applied impact energy.

A rearrangement of the diagram's axis to peak impact force (Fm) versus runner body mass provides refined information as to the cushioning capacity of the midsole system. By considering impact velocity as a further variable (as demonstrated in Figure 4(b)), the results yield performer-specific insight. To better interpret body mass-dependent results, some benchmark values concerning anthropometric data have to be considered as outlined in Figure 4(b). This information reflects a female runner with a EUR 38 shoe size, who is expected to have an average height of 169 cm [20]. As normal BMI values range from 18.5 to 24.9, she should thus normally weigh between 52.2 and 70.3 kg, whereas a female athlete of a 19.7 BMI would weigh closer to the lower end of this range at 55.6 kg.

According to these results, a runner with a body mass of around 60 kg is exposed (at each foot strike) to an impact cushioned by about 30 kg less when compared to a 10 kg lighter runner. In a similar fashion, runners exceeding a 65 kg barrier are not as well accommodated by the shoe as the athletes, who should, according to anthropometric data (shoe size and gender), fall close to the optimum weight range of the midsole's cushioning properties.

As kinetic impact energy is the product of mass multiplied by the square of its velocity, an increase in vertical impact speed from 0.4 to 0.5 m/s should result in a shock wave augmentation of 64%. This is equivalent to a runner gaining 64% in body mass while running at the same speed. The tested midsole material was indeed slightly strain-rate sensitive, in terms of force attenuation, as its rapid rate force-cushioning characteristics seem to improve as the impact velocity increased. This would, counterintuitively, suggest that the gel-based cushioning system is more efficient in mitigating the occurring shock during running than jogging.

The shock attenuation properties of the midsole strongly depend on the stiffness (Sm) of the compound midsole material (most midsoles comprise more than one material). This stiffness is defined as the ratio of peak force to maximum displacement (Dm).

(1) $$\begin{array}{c} Sm=\frac{Fm}{Dm}. \end{array}$$

**Figure 5:**
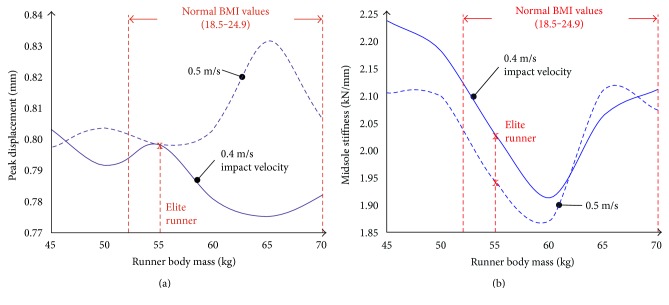
Runner body mass-dependent (a) displacement at peak force and (b) average midsole stiffness.

The values of the recorded displacement, at peak force, are summarized in Figure 5(a) with respect to the runner's body mass and strike velocity, whereas the average stiffness of the midsole material, calculated according to (1), is shown in Figure 5(b) as a function of the same variables. According to the data presented in Figure 5(b), the shoe stiffness is comparable to that of a human leg; thus, peak displacement values demonstrated in Figure 5(a) can be used to evaluate the shoe with respect to the absorbed energy; that is, low stiffness is expected to provide better cushioning at higher displacement values.

It is noteworthy that the measured displacement is affected by the loading rate of the midsole. A closer look however at both Figure 5(a) and Figure 5(b) reveals that the midsole system is exceptionally well engineered regarding elite runners, as the specific body mass value (presumed for athletes) seems to provide strain rate-independent displacement values. The midsole stiffness, being at both loading rates close to the shoe's optimum, should thus provide excellent cushioning at low and constant displacement values, accommodating athletes with a constant and superior stability when compared to any other body mass range.

Strain (*ε*), described by (2), is indicative of the shock attenuation response of a midsole of thickness (*B*) when subjected to a compressive force resulting in a displacement.

(2) $$\begin{array}{c} \varepsilon =\frac{Dm}{B}. \end{array}$$

The strain, occurring during the impact, was calculated by the displacement values recorded during peak force. As the artificial heel morphology was graded in 5 consecutive levels to simulate a foot strike with a realistic plantar pressure distribution (see Figure 1), each level induced a strain corresponding to the offset value of the initial heel surface and the average midsole thickness at the heel-shoe contact. Average strain values calculations were based on the contribution of each level to the total impact surface, while peak strain corresponded to the maximum calculated strain among all levels. The mean values and deviations of average and peak strain as calculated for all measurements are summarized in Table 1, for both impact velocities.

**Figure 6:**
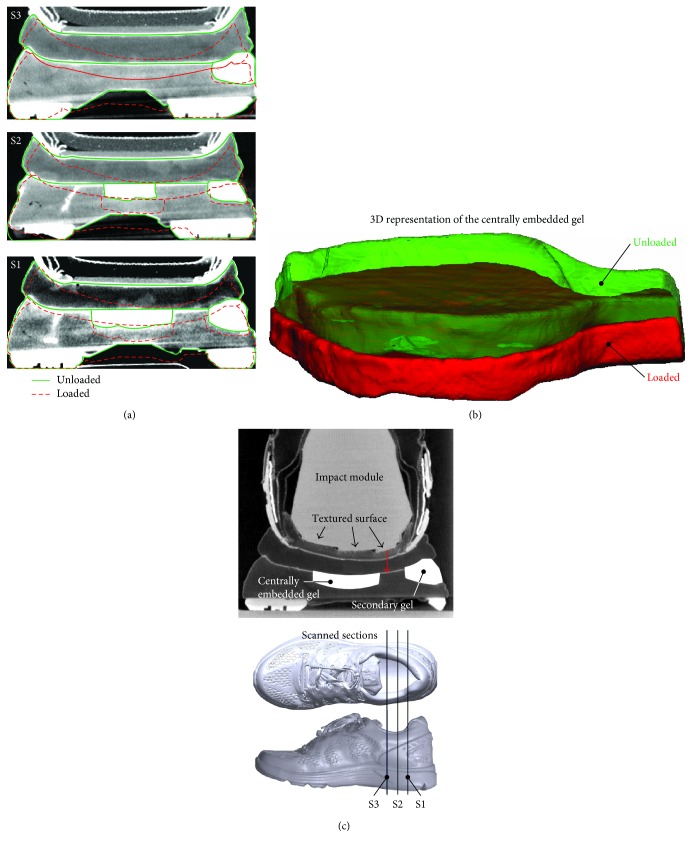
(a) Body mass-specific displacement profile at different anterior-posterior cross sections of the shoe. (b) 3D deformation of the central gel. (c) Off-axis positioning of the embedded gel (as for normal pronation).

Deformation and stability of the footwear system were further evaluated by in situ loading of the midsole-insole system in a *μ*CT device. The 3D displacement profiles were analysed, as shown in Figure 6(a), and the stability of the shoe was evaluated in terms of the vertical to transverse displacement ratio. Although the overall volume of the centrally positioned gel did not change during compression, both its position and its geometry were altered significantly in response to the heel impact. This deformation, demonstrated in Figure 6(b) through two superimposing 3D gel structures (green being undeformed and red corresponding to the loaded one), is in line with the centre of gravity of the impacting module.

The measurements showed that low-impact energies, corresponding to a runner body mass of about 60 kg, had a marginal effect on shoe stability, although the absolute values of both transverse and anteroposterior deformation increased. Higher body masses resulted in deformations exhibiting higher values in the transverse axis than in the anteroposterior one. This led to a significant decrease in shoe stability beyond deformations corresponding to a 60 kg runner, a response which is likely to result due to the off-axis loading of the midsole's energy-dissipating mechanism (embedded gel), as designated by the red arrow in Figure 6(c), and a decrease in midsole stiffness beyond this body mass (Figure 5(b)).

## 4. Discussion and Implications

The results of this study emphasize on the importance of the runner body mass range that fully utilizes the properties of a specific cushioning system, as this can dramatically influence the impact forces experienced by an athlete or habitual runner. Hamill et al. [27] stipulated that foot impact is attenuated passively at the articular cartilage, ligaments, and heal pad as it passed through the runner's musculoskeletal system, a statement which is in line with injury epidemiology of long-distance runners. As illustrated in Figure 4(b), a female runner weighing 50 kg and wearing the shoe type/size tested would be exposed, at every stride, to a shock wave increased by almost 300 N when compared to a runner weighing 58–62 kg. She would thus be better accommodated with another cushioning system or a midsole with a higher gel concentration or different distribution.

To provide an estimate of how this load increase is transferred to the cartilaginous tissue of the knee joint, both the surface area of the medial tibial plateau, about 1670 mm^2^ [28], and the time to peak force during running (which is on the order of 30 ms) have to be considered. This roughly translates in a stress augmentation of ~0.1 MPa within the knee joint (from approximately 0.8 to 0.9 MPa), at a stress rate of 30 MPa/s. Onset damage is expected to occur at higher stress values, of about 3–6 MPa [29], and loading rates exceeding 1000 MPa/s [30]. Recent literature, however, stipulates that cartilaginous tissue exposed to lesser stress values is prone to a delayed biological response [31], and thus, even an increase of this magnitude over the duration of a 42 km marathon could indeed result in cell apoptosis.

Velocity driven impacts, as described in Procedure A of ASTM F1614–99, allow the determination of strain rate-dependent properties which were shown to exhibit, in this case, an important effect only on shoe stability. This is expected to be even more pronounced in other midsole technologies, where the force application rate is likely to also alter the shock attenuation capacity of the shoe. The effect of the impulse should therefore be carefully considered.

Recent studies have shown that even recreational runners can observe changes in midsole stiffness greater than 15 kN/m [32], and while most runners are able to quickly identify footwear as comfortable or not [33], even elite athletes fail to correctly asses the magnitude of the impact peak in the ground reaction force. This is in line with Gibson's [34] notion, disregarding passive perception during locomotion. Gibson argued an information-based perception of the environment that invariants are extracted over time rather than being conveyed by the nervous system. Engaging this theorem, Kim et al. [9] recently associated leg stiffness to the running environment. Based on our results, we would stress the importance of considering the midsole's cushioning capacity as a significant subcomponent of this performer-environment system.

Even though the perception of comfort can be modulated through the material selection of a midsole system, it is not the only factor considered during the development of athletic footwear, which was to provide proper stability during the foot-ground contact. As per results of this study, stiffness of the examined midsole system strongly depends on the runner's body mass, an effect that was less pronounced for higher loading rates.

Cushioning systems found in athletic footwear are also susceptible to several other parameters next to strike pattern, impact speed, and runner body mass. Loading history, exposure to elevated temperatures is a parameter that significantly alters the shock attenuation properties of the footwear [35] and is thus an important criterion during the selection of a running shoe, as the same shoe would perform differently for the same athlete when worn in cold versus warm climates.

Even though the purpose of this study was not to evaluate a specific shoe but to provide insight on aspects that could render a footwear appropriate or not for individuals, the results of this investigation might be subject to variations in shoe type and even size. Further limitations of this study revolve around the different strike patterns that a shoe might be exposed to. Even though the cushioning capacity of the midsole system would not change based on pronation type, it is likely to slightly alter the force dissipation due to different plantar pressure distributions. Kim et al. [9] recently suggested that leg stiffness is related to the running environment, thus indicating that underfoot cushioning would be perceived differently even among performers with similar strike patterns. Mid- and forefoot strikers are also expected to be exposed to impact cushioned differently to what is predicted here, although footwear design considers midsole thickness-dependent foot strike in terms of actively loaded midsole surface, for example, heel strikers may benefit from an increased midsole thickness when compared to forefoot strikers but lack in foot-ground contact surface, thus resulting in a similar ration of strike pressure to midsole thickness.

## 5. Conclusion

The initial hypothesis of this study was confirmed, as body mass was found to significantly influence the capacity of technical footwear to cushion impulsive forces generated during running.

Counterintuitively, heavier runners are not necessarily exposed to higher impacts than the lighter ones. The selection of a midsole system optimized for a specific weight range is thus vital in reducing the likelihood of joint overloading during long-distance running, which is known to elevate proinflammatory cytokine production [36]. Athletes and recreational runners could in this way prolong the duration of physical exercise beneficial to maintaining a simulated immune system, in favour of anabolic over catabolic activity.

Although modern footwear is capable of properly attenuating the shock occurring during foot strike, athletic shoes should be, in conclusion, carefully chosen based on subject body mass and the intended activity.

## Figures and Tables

**Table 1:** Runner body mass-dependent strain (peak and average) of the tested midsole system.

Runner body mass (kg)	Peak strain				Average strain			
	0.4 m/s loading rate		0.5 m/s		0.4 m/s		0.5 m/s	
	Mean	Deviation	Mean	Deviation	Mean	Deviation	Mean	Deviation
45	0,2636	10^−7^	0,2636	3×10^−7^	0,0868	1×10^−7^	0,0868	3×10^−7^
50	0,2643	9×10^−7^	0,2636	0,0	0,0874	8×10^−7^	0,0868	0,0
55	0,2633	15×10^−7^	0,2632	3×10^−7^	0,0865	13×10^−7^	0,0865	2×10^−7^
60	0,2631	2×10^−7^	0,2635	3×10^−7^	0,0863	2×10^−7^	0,0867	3×10^−7^
65	0,2633	1×10^−7^	0,2634	3×10^−7^	0,0865	1×10^−7^	0,0866	1×10^−7^
70	0,2635	7×10^−7^	0,2640	5×10^−7^	0,0867	6×10^−7^	0,0872	4x10^−7^
